# Angiotensin II blockade had no effect on range of motion after total knee arthroplasty: a retrospective review

**DOI:** 10.1186/s13018-020-1555-5

**Published:** 2020-02-12

**Authors:** Joseph R. Langston, Duncan C. Ramsey, Kathryn Skoglund, Kathryn Schabel

**Affiliations:** Oregon Health and Science University, 3181 SW Sam Jackson Park Road, Mail Code OP31, Portland, OR 97239 USA

**Keywords:** Angiotensin, Total knee arthroplasty, Range of motion, ACE inhibitors, ARBs, Flexion, Extension

## Abstract

**Background:**

Stiffness and pain from arthrofibrosis following total knee arthroplasty (TKA) is a challenging problem, and investigating methods to prevent or reduce the incidence of postoperative arthrofibrosis is critical. Studies have shown that angiotensin-converting enzyme inhibitors (ACEIs) and angiotensin receptor blockers (ARBs) are efficacious at preventing fibrotic disorders in the lungs, liver, kidneys, and eyes. Our aim was to determine if ACEI or ARB use postoperatively reduces the incidence of arthrofibrosis in TKA patients.

**Methods:**

In a retrospective review, we analyzed 141 consecutive TKAs performed at a single institution by a single surgeon from December 2010 to December 2014. Range of motion (ROM) in patients already taking ACEI, ARB, or neither medication was compared. Independent variables recorded were gender, age, BMI, presence of diabetes or preoperative opioid or statin use, preoperative ROM, and use of ACEIs or ARBs. Dependent variables recorded were postoperative knee flexion, extension, and total arc of motion. The primary outcome variable was success or failure of achieving 118^o^ total arc of motion postoperatively, based on a study that found significant compromise of function in TKA patients who failed to obtain this goal. Secondary endpoints were postoperative knee flexion, extension, and total arc of motion.

**Results:**

The use of neither ACEIs nor ARBs showed a significant difference in attaining greater than 118° of motion postoperatively compared to controls at 6 months. Significant predictors of obtaining > 118° motion were BMI (*p* < 0.05), preoperative flexion (*p* < 0.001), and preoperative total arc of motion (*p* < 0.002). Significant predictors of secondary ROM outcomes were preoperative ROM and BMI.

**Conclusions:**

Our study demonstrated that the principle predictor of postoperative ROM is BMI and preoperative ROM. The use of ACEIs or ARBs did not result in a greater likelihood of obtaining satisfactory ROM postoperatively.

## Background

Total knee arthroplasty (TKA) is an effective treatment option for eliminating knee pain, restoring mobility, and improving the overall quality of life for patients with end-stage knee arthritis. Given the general success of meeting these goals, the procedure is becoming more popular among patients of all age groups [1–3]. In 2010, there were approximately 720,000 knee replacements performed, with an estimated 4 million adults in the USA currently living with a total knee replacement [4, 5]. Based on historical data, projections of future demand for primary TKA are expected to reach 3.48 million annually by 2030 [6]. Despite the overwhelmingly positive outcomes of TKA for end-stage osteoarthritis, 15–20% of patients remain dissatisfied secondary to stiffness or pain [7–10].

There are no universally accepted criteria for stiffness or the need for intervention; however, certain motion requirements are known for activities of daily living. A quantitative analysis of knee motion has shown that patients require 67° of knee flexion during the swing phase of gait, 83° to ascend stairs, 90° to 100° to descend stairs, 93° to rise from a standard chair, and up to 105° to rise from a low chair [11]. Patients with poor functional knee range of motion after TKA with adequate component alignment, sizing, and position are typically treated with manipulation under anesthesia within the first 3 months postoperatively or treated for scar revision/lysis of adhesions after 3 months [12–15]. Both procedures are costly and cause delays to the overall recovery process. Ritter et al. [16] showed that functional outcome and Knee Society Scores became significantly compromised with a total arc of motion of less than 118°.

Postoperative wound healing and scar formation are complex processes involving a sophisticated network of immune cells, cell mediators, and inflammatory cytokines. The formation of fibrous tissue is regulated by the transcriptional activation of collagen gene expression. Transforming growth factor Beta-1 (TGF-β1) is a crucial regulator of this process [17–20]. Working to control the expression of extracellular matrix components as well as the expression of protease inhibitors, TGF- β1 has a combined anabolic and anti-catabolic effect on tissue fibrosis [21]. TGF-β1 is also a central cytokine that has been linked in the signaling pathway for the differentiation of myogenic precursor cells into myofibroblasts in skeletal muscle [22]. Similarly, upregulation and overexpression of TGF-β1 have been shown to increase the production of myofibroblasts and the propagation of fibrosis in lung disease [23, 24], liver cirrhosis [25], cardiac injury [26], and renal fibrosis [27].

One of the mechanisms by which TGF-β1 expression increases is through the action of the main end product of the renin-angiotensin-system (RAS), angiotensin II [28]. Although primarily known for their role in regulating blood pressure, components of the RAS axis, such as angiotensin-converting enzyme and angiotensin 1 receptor, have been found in increased levels in fibrotic skeletal muscle dystrophies [29]. In fact, angiotensin receptor blockers (ARBs) have been used to successfully decrease fibrosis and improve skeletal muscle features in dystrophic muscle [30, 31]. ACE inhibitors (ACEIs) and ARBs are used as standard of care in preventing cardiac fibrosis in the setting of heart failure or post-myocardial infarction and in preventing renal fibrosis in the setting of renal disease or diabetes.

Many patients undergoing total knee arthroplasty are taking ACEIs inhibitors or ARBs for hypertension, coronary artery disease, congestive heart failure management or diabetes. We hypothesized that patients taking ACEIs or ARBs will have improved postoperative range of motion (ROM) compared to controls. Exploration of this potential relationship between ACE inhibitor or ARB use and improved range of motion after total knee arthroplasty has the potential to improve patient functional outcomes and satisfaction. The prevalence of stiffness after primary TKA has been reported to range between 1.3 and 5.3% [32, 33], and incidence of manipulation under anesthesia after primary TKA has been reported as ranging from 1.5 to 3.8% [25, 26]. With so many procedures performed annually, the number of patients suffering from this complication is substantial and will continue to grow.

## Methods

### Patient population

We examined data from all primary TKAs performed by a single attending surgeon (KS) from December 2010 to December 2014. Institutional review board approval was obtained. The study population assessed for eligibility consisted of 325 patients with primary TKA in 373 knees.

For inclusion in the study, patients were a minimum of 18 years of age, had a follow-up visit at least 180 days postoperatively, and charts must have indicated preoperative knee flexion, extension, and total arc of motion. Study exclusion criteria were patients undergoing revision TKA, unicompartmental knee arthroplasty, patients who developed postoperative infection, or patients lost to follow-up. Patients were not excluded if they underwent prior arthroscopy, as this has not been shown to affect postoperative range of motion after TKA [12].

If the patients used nicotine, they were required to stop using nicotine products a minimum of 6 weeks prior to their TKA. Dose ranges for all medications were within specifications for hypertension, heart failure, coronary artery disease, and diabetes. All surgeries were conducted using a tourniquet inflated to 250 mmHg. All motion measurements were made using a goniometer during pre-operative and follow-up clinic visits. Measurements were made by the primary investigator (KS).

### Outcomes

Independent variables recorded were gender, age, BMI, presence of diabetes or preoperative opioid or statin use, preoperative knee flexion, extension, and total arc of motion, and use of ACEIs or ARBs (Table 1). Dependent variables recorded were postoperative knee flexion, extension, and total arc of motion. The primary outcome variable was success or failure of achieving 118^o^ total arc of motion postoperatively, based on a study that found significant compromise of function in TKA patients who failed to obtain this goal [16]. Secondary endpoints were measurements of knee flexion, extension, and total arc of motion. In order to assess differences in the magnitude of improvement after surgery, the difference in pre- versus postoperative flexion, extension, and total arc of motion between groups was also compared.

**Table 1:** Demographic and preoperative characteristics

	ACEIs (*N* = 35)	ARBs (*N* = 19)	Controls (*N* = 87)	*p* value
Male, %	19	6	27	0.048^a^
Age, years; mean ± SD	63.6 ± 7.9	66.4 ± 7.8	61.5 ± 10.0	1.710^b^
Comorbidities				
BMI, kg/m^2^	33.7 ± 6.7	31.1 ± 6.5	30.4 ± 6.7	0.050^c^
Diabetes, *N*	10	6	5	< 0.001^a^
Opioids, *N*	17	5	31	0.226
Statin, *N*	18	10	21	0.004^a^
Preoperative ROM, degrees; mean ± SD				
Flexion	107.7 ± 13.7	108.9 ± 11.6	111.0 ± 12.3	0.423^b^
Extension	3.1 ± 3.8	2.4 ± 4.5	2.3 ± 3.9	0.366^b^
Total arc	104.6 ± 15.9	106.5 ± 13.3	108.7 ± 13.8	0.317^b^
Manipulation under anesthesia	2	1	5	1.000^d^

*SD* standard deviation, *BMI* Body mass index, *ROM* Range of motion

Demographic data for study patients analyzed. ^a^*χ*^*2*^, ^b^Kruskal, ^c^ANOVA, ^d^Fisher exact

The primary groups of interest were patients taking ACEIs versus those that were not. Given the similarity of the mechanism of action of ACEIs and ARBs, separate analyses were also conducted comparing outcomes of patients taking ARBs versus those that were not as well as analysis comparing patients on ACEIs or ARBs versus those not taking either (controls).

There were eight additional patients with a single TKA that required manipulation under anesthesia. We excluded these patients from the analysis of primary and secondary endpoints but did compare the frequency of this outcome based on ACE inhibitor and/or ARB.

### Data analysis

Demographic and clinical variables were evaluated against primary and secondary endpoints using *χ*^*2*^, Fisher’s exact test, Spearman’s correlation, the Mann-Whitney test, or logistic regression as appropriate. The Shapiro-Wilk test was used to assess variable distributions for normality. To account for any potential confounding, the primary outcome was also evaluated with patients stratified by age (greater or less than 65 years), gender, and whether they carried a diagnosis of diabetes mellitus. A post hoc power analysis was done on the primary variable (whether 118° total arc of motion was attained) to determine the effect size that the *χ*^*2*^ test would be able to discern with the sample size of 141 knees that met our inclusion criteria.

For patients that underwent bilateral TKAs, treating both knees as independent patients violates statistical independence upon which many statistical tests are based. There is substantial variability in the orthopedic literature concerning the statistical analysis of bilateral surgery in a single patient. A meta-analysis of all original articles using bilateral cases that had been published in The Journal of Bone and Joint Surgery (American Volume) over a 2-year period revealed that 25% of the published literature were found to have possibly violated statistical independence [34]. Given this concern, outcome analyses were repeated using generalized estimating equations (GEEs). These methods are able to account for repeated data points from the same patient. Results using GEEs were then compared to the original results in order to evaluate whether the violation of independence introduced errors.

## Results

The study population consisted of 376 knees that underwent TKA in 325 patients. Of these, 274 patients had a single TKA, 46 patients had consecutive bilateral TKAs, and 5 patients had simultaneous bilateral TKAs. Three knees from this dataset were excluded due to the development of prosthetic joint infection and 224 knees were excluded due to follow-up less than 180 days postoperatively. There were eight additional patients with a single TKA that required manipulation under anesthesia. We excluded these patients from the overall statistical analysis but did compare the frequency of this outcome based on ACEIs and/or ARB. Therefore, postoperative range of motion data from 141 TKA’s was analyzed.

Table 1 shows demographic data and preoperative characteristics for the group taking ACEIs, the group taking ARBs, and the control group that was not on ACEIs or ARBs. Age did not differ significantly between groups; however, there was a group difference based on gender (*p* = 0.048). Comorbidities and preoperative range of motion did not differ significantly between groups.

Mean (±SD) postoperative total arc of motion for patients taking ACEIs, ARBs, and controls was 114.8° ± 12.7°, 115.6° ± 10.7°, and 115.6° ± 12.5°, respectively, and these values did not differ significantly. Therefore, the primary outcome, success or failure of achieving 118^o^ total are of motion, was not affected by ACEI or ARB use.

Table 2 shows the degrees of improvement in motion measures after TKA in patients taking ACEIs, ARBs, and in controls. Improvement in flexion, extension, and total arc of motion was not significantly different between the groups.

**Table 2:** Improvement in range of motion measures (in degrees) after total knee arthroplasty

	ACEIs	No ACEIs	*p* value
Flexion	9.86	8.44	0.563^a^
Extension	0.23	0.95	0.549^b^
Total arc	10.20	7.28	0.417^a^
	ARB	No ARB	*p* value
Flexion	8.16	8.89	0.856^a^
Extension	1.32	0.70	0.774^b^
Total arc	9.05	7.85	0.777^a^
	ACEI or ARB	Controls	*p* value
Flexion	9.26	8.51	0.745^a^
Extension	0.61	0.87	0.742^b^
Total arc	9.82	6.90	0.266^a^

Statistical comparisons between groups were made using: ^a^Student’s *t* test; ^b^Kruskal

Analyses done combining ARB with ACEI patients did not show any significant difference with respect to the primary endpoint, failure to achieve 118^o^ total arc of motion (*p* = 0.95 for ACEI, *p* = 0.48 for ARB, *p* = 0.46 for ARB + ACEI group) nor to secondary endpoints of knee flexion, extension, and total arc of motion (Table 3). Stratification by age, BMI, gender, and having diabetes also did not show significant differences in outcomes between the different medication groups.

**Table 3:** Predictors of final ranges of motion measures (*p* values)

	Obtaining 118^○^ motion	Postoperative flexion	Postoperative extension	Postoperative total arc
Male	0.911	0.161	0.666	0.580
Age	0.429	0.518	0.551	0.955
BMI	0.055	*< 0.001*	0.208	*0.004*
Diabetes	0.656	0.164	0.431	0.410
ACEI use	0.948	0.543	0.749	0.884
ARB use	0.481	0.594	0.677	0.929
Preoperative range of motion				
Flexion	*0.001*	*< 0.0001*	0.055	*< 0.001*
Extension	0.448	0.854	*0.048*	0.350
Total arc	*0.002*	*< 0.001*	*0.037*	*< 0.001*

Statistically significant *p*-values are italicized

The post hoc power analysis revealed that with 141 patients, a significance level of 0.05, and a power of 80%, the *χ*^*2*^ test would discern an effect size of 0.24. Commonly accepted definitions hold that for *χ*^*2*^ tests, an effect size of 0.1 is a small effect size, and 0.3 is a medium effect size.

Several independent variables were significant predictors of postoperative range of motion (Table 3). Again, ACEI or ARB use was not predictive of range of motion. However, BMI was a significant predictor of postoperative flexion (*p* < 0.001) and total arc of motion (*p* = 0.004). Preoperative flexion was a significant predictor of postoperative flexion (*p* = 0.001) and total arc of motion (*p* < 0.001), as well as a significant predictor of obtaining 118^o^ total arc of motion (*p* = 0.001). Total arc of motion preoperatively was a significant predictor of all postoperative motion parameters measured, and preoperative extension was a predictor of postoperative extension (*p* = 0.048). Neither ACEI nor ARB use was predictive of obtaining 118^o^ when stratified by gender, diabetes mellitus, or age (greater than versus less than 65). Rates of manipulation under anesthesia similarly did not differ significantly between the groups (Table 1).

## Discussion

Restoration of functional ROM is a crucial goal of knee arthroplasty, and failure to do so results in increased dissatisfaction among patients. While there are many causes of dissatisfaction, ROM is one of the few objective findings that can be followed postoperatively. Our study found no significant difference in postoperative ROM between patients taking ACEIs or ARBs versus controls taking neither medication. While many other clinical and animal model studies have demonstrated decreased fibrosis after angiotensin II blockade [23, 24, 26–28], oral administration of clinical doses for common indications of hypertension, coronary artery disease, congestive heart failure, or diabetes did not show an effect on range of motion after total knee arthroplasty in our study population. To our knowledge, this is the first study to have explored this association.

Regarding secondary outcome measures, significant predictors of obtaining satisfactory ROM include BMI and preoperative ROM. These findings reinforce data from other studies on ROM after total knee arthroplasty. A retrospective review of 391 consecutive total knee arthroplasties found that patients with higher BMIs had lower pre- and postoperative ROM as well as higher rates of manipulation under anesthesia [35]. A similar review of 135 patients who underwent TKA evaluated whether specific pre- and postoperative variables were correlated with postoperative ROM [36]. Their results demonstrated that preoperative ROM was the only significant predictor of postoperative ROM.

Ritter et al. [37] retrospectively reviewed more than 4700 total knee arthroplasty surgeries using regression tree analysis to characterize the combinations of variables influencing the postoperative ROM. The principal predictive factor of the postoperative ROM in that study was the preoperative ROM. Other factors that were significantly associated with reduced flexion were intraoperative flexion, gender, preoperative tibiofemoral alignment, age, and posterior capsular release. No correlation of BMI to knee ROM was found in Ritter’s study; however, this study endpoint was 3 months from surgery as opposed to our 6-month endpoint.

One possible explanation for our findings of ACEIs and ARBs not having an effect on postoperative ROM is that patients taking those medicines were more likely to have greater BMIs. Given that more obese patients are more likely to have worse motion [35], this could have confounded our results.

Another explanation could be limitation by dosing administration. ACEIs and ARBs are all taken orally, and it is unclear exactly how much intraarticular permeation takes place with these medicines. It is also unclear exactly what dose would be required to optimize the anti-fibrotic effects of these drugs, and it is possible that the standard dosing for hypertension, as was the case for our patients, falls below that threshold.

Analysis taking into account the repeated measures of patients who underwent TKA on both knees did not yield different results from analysis treating all TKAs as independent trials with respect to the primary outcome. While this could lead to a violation of statistical independence, in theory, our data supports similar findings in previous studies [38–40] suggesting that bilaterality may be excluded in studies on TKA outcomes.

One limitation of this study is that all surgeries and measurements were done by a single surgeon. In addition, roughly 60% of patients in the original dataset were excluded, primarily for insufficient follow-up (> 180 days). The introduction of selection bias is likely not substantial, however, as patients excluded for this reason did not differ from the rest of the dataset with respect to demographic or clinical variables, including preoperative ROM.

A second limitation may be that there was a large variability with respect to the length of postoperative follow-up in patients assessed for study eligibility, with a median of 118 days (range 11–1507 days). Ritter et al. have previously demonstrated that clinically significant ROM gained after TKA plateaus after roughly 3 months [37]. However, preliminary analysis showed that patients with postoperative follow-up of greater than 180 days had significantly improved ROM in terms of the primary as well as secondary endpoints compared with patients with less than 180 days of follow-up. This reinforced our inclusion criteria of at least 180 days of follow-up in order to capture the true ROM attained by patients.

Given the data from studies in medical disciplines and the promise of prevention or reversal of fibrotic pathology, future study of the effect of these medicines is warranted in knee arthroplasty patients. This could include randomized controlled trials with large numbers of patients in each arm of the study using either oral or locally injected forms of the medications.

## Conclusions

We conclude that the principle predictor of postoperative ROM is BMI and preoperative ROM. The use of ACEIs or ARBs did not result in a greater likelihood of obtaining satisfactory ROM postoperatively. Finally, we showed that bilaterally may be ignored as each knee has statistical independence. As the literature continues to show a significant number of patients dissatisfied after TKA and range of motion being of paramount importance after TKA, these results will help guide future research into the question of how to improve outcomes.

## Data Availability

The datasets analyzed during the current study are available from the corresponding author on reasonable request.

## Abbreviations

ACEI: Angiotensin-converting enzyme inhibitors
ARBs: Angiotensin receptor blockers
GEEs: Generalized estimating equations
RAS: Renin-angiotensin-system
ROM: Range of motion
TGF-β1: Transforming growth factor beta-1
TKA: Total knee arthroplasty

## References

[1] Jain NB, Higgins LD, Ozumba D, Guller U, Cronin M, Pietrobon R, Katz JN (2005). Trends in epidemiology of knee arthroplasty in the United States, 1990-2000. Arthritis Rheum.

[2] Kim S (2008). Changes in surgical loads and economic burden of hip and knee replacements in the US: 1997-2004. Arthritis Rheum.

[3] Singh JA (2011). Epidemiology of knee and hip arthroplasty: a systematic review. Open Orthop J.

[4] Centers for Disease Control and Prevention / National Hospital Discharge Survey. *Number of all-listed procedures for discharges from short stay hospitals, by procedure category and age: United States, 2010*. http://www.cdc.gov/nchs/data/nhds/4procedures/2010pro4_numberprocedureage.pdf. Accessed 20 Nov 2019.

[5] Weinstein AM, Rome BN, Reichmann WM, Collins JE, Burbine SA, Thornhill TS (2013). Estimating the burden of total knee replacement in the United States. J Bone Joint Surg Am.

[6] Kurtz S, Ong K, Lau E, Mowat F, Halpern M (2007). Projections of primary and revision hip and knee arthroplasty in the United States from 2005 to 2030. J Bone Joint Surg Am.

[7] Jacobs CA, Christensen CP (2014). Factors influencing patient satisfaction two to five years after primary total knee arthroplasty. J Arthroplast.

[8] Matsuda S, Kawahara S, Okazaki K, Tashiro Y, Iwamoto Y (2013). Postoperative alignment and ROM affect patient satisfaction after TKA. Clin Orthop Relat Res.

[9] Ali A, Sundberg M, Robertsson O, Dahlberg LE, Thorstensson CA, Redlund-Johnell I (2014). Dissatisfied patients after total knee arthroplasty: a registry study involving 114 patients with 8-13 years of follow-up. Acta Orthop.

[10] Kim TK, Chang CB, Kang YG, Kim SJ, Seong SC (2009). Causes and predictors of patient's dissatisfaction after uncomplicated total knee arthroplasty. J Arthroplast.

[11] Laubenthal KN, Smidt GL, Kettelkamp DB (1972). A quantitative analysis of knee motion during activities of daily living. Phys Ther.

[12] Cheuy VA, Foran JRH, Paxton RJ, Bade MJ, Zeni JA, Stevens-Lapsley JE (2017). Arthrofibrosis associated with total knee arthroplasty. J Arthroplast.

[13] Thompson R, Novikov D, Cizmic Z, Feng JE, Fideler K, Sayeed Z (2019). Arthrofibrosis after total knee arthroplasty: pathophysiology, diagnosis, and management. Orthop Clin North Am.

[14] Schiavone Panni A, Cerciello S, Vasso M, Tartarone M (2009). Stiffness in total knee arthroplasty. J Orthop Traumatol.

[15] Bong MR, Di Cesare PE (2004). Stiffness after total knee arthroplasty. J Am Acad Orthop Surg.

[16] Ritter MA, Lutgring JD, Davis KE, Berend ME (2008). The effect of postoperative range of motion on functional activities after posterior cruciate-retaining total knee arthroplasty. J Bone Joint Surg Am.

[17] Kim KK, Sheppard D, Chapman HA. TGF-β1 Signaling and tissue fibrosis. Cold Spring Harb Perspect Biol. 2018;10(4):a022293. 10.1101/cshperspect.a022293 PMC5880172 28432134

[18] Meng XM, Nikolic-Paterson DJ, Lan HY (2016). TGF-β: the master regulator of fibrosis. Nat Rev Nephrol.

[19] Clark DA, Coker R (1998). Transforming growth factor-beta (TGF-beta). Int J Biochem Cell Biol.

[20] Lawrence DA (1996). Transforming growth factor-beta: a general review. Eur Cytokine Netw.

[21] Verrecchia F, Mauviel A, Farge D (2006). Transforming growth factor-beta signaling through the Smad proteins: role in systemic sclerosis. Autoimmun Rev.

[22] Li Y, Huard J (2002). Differentiation of muscle-derived cells into myofibroblasts in injured skeletal muscle. Am J Pathol.

[23] Khalil N, O'Connor RN, Unruh HW, Warren PW, Flanders KC, Kemp A (1991). Increased production and immunohistochemical localization of transforming growth factor-beta in idiopathic pulmonary fibrosis. Am J Respir Cell Mol Biol.

[24] Koli K, Myllärniemi M, Keski-Oja J, Kinnula VL (2008). Transforming growth factor-beta activation in the lung: focus on fibrosis and reactive oxygen species. Antioxid Redox Signal.

[25] Weng H, Mertens PR, Breitkopf K, Gressner AM, Dooley S (2005). IFN-γ abrogates profibrogenic TGF-β signaling in the liver by targeting the expression of pathway-restricted and inhibitory Smads. Z Gastroenterol.

[26] Dobaczewski M, Chen W, Frangogiannis NG (2011). Transforming growth factor (TGF)-β signaling in cardiac remodeling. J Mol Cell Cardiol.

[27] Wang B, Jha JC, Hagiwara S, McClelland AD, Jandeleit-Dahm K, Thomas MC (2014). Transforming growth factor-β1-mediated renal fibrosis is dependent on the regulation of transforming growth factor receptor 1 expression by let-7b. Kidney Int.

[28] Morales MG, Vazquez Y, Acuña MJ, Rivera JC, Simon F, Salas JD (2012). Angiotensin II-induced pro-fibrotic effects require p38MAPK activity and transforming growth factor beta 1 expression in skeletal muscle cells. Int J Biochem Cell Biol.

[29] Sun G, Haginoya K, Dai H, Chiba Y, Uematsu M, Hino-Fukuyo N (2009). Intramuscular renin-angiotensin system is activated in human muscular dystrophy. J Neurol Sci.

[30] Cohn RD, van Erp C, Habashi JP, Soleimani AA, Klein EC, Lisi MT (2007). Angiotensin II type 1 receptor blockade attenuates TGF-beta-induced failure of muscle regeneration in multiple myopathic states. Nat Med.

[31] Cabello-Verrugio C, Morales MG, Cabrera D, Vio CP, Brandan E (2012). Angiotensin II receptor type 1 blockade decreases CTGF/CCN2-mediated damage and fibrosis in normal and dystrophic skeletal muscles. J Cell Mol Med.

[32] Nelson CL, Kim J, Lotke PA (2005). Stiffness after total knee arthroplasty. J Bone Joint Surg Am.

[33] Yercan HS, Sugun TS, Bussiere C, Selmi TAS, Davies A, Neyret P (2006). Stiffness after total knee arthroplasty: prevalence, management and outcomes. Knee.

[34] Park MS, Kim SJ, Chung CY, Choi IH, Lee SH, Lee KM (2010). Statistical consideration for bilateral cases in orthopaedic research. J Bone Joint Surg Am.

[35] Gadinsky NE, Ehrhardt JK, Urband C, Westrich GH (2011). Effect of body mass index on range of motion and manipulation after total knee arthroplasty. J Arthroplast.

[36] Gatha NM, Clarke HD, Fuchs R, Scuderi GR, Insall JN (2004). Factors affecting postoperative range of motion after total knee arthroplasty. J Knee Surg.

[37] Ritter MA, Harty LD, Davis KE, Meding JB, Berend ME (2003). Predicting range of motion after total knee arthroplasty. Clustering, log-linear regression, and regression tree analysis. J Bone Joint Surg Am.

[38] Na YG, Kang YG, Chang MJ, Chang CB, Kim TK (2013). Must bilaterality be considered in statistical analyses of total knee arthroplasty?. Clin Orthop Relat Res.

[39] Schwarzer G, Schumacher M, Maurer TB, Ochsner PE (2001). Statistical analysis of failure times in total joint replacement. J Clin Epidemiol.

[40] Robertsson O, Ranstam J (2003). No bias of ignored bilaterality when analysing the revision risk of knee prostheses: analysis of a population based sample of 44,590 patients with 55,298 knee prostheses from the national Swedish knee Arthroplasty register. BMC Musculoskelet Disord.

